# Thyroid malignancy in children: where does it locate?

**DOI:** 10.20945/2359-3997000000603

**Published:** 2023-05-10

**Authors:** Hasibe Gokce Cinar, Cigdem Uner, Ozlem Kadirhan, Sonay Aydin

**Affiliations:** 1 Dr. Sami Ulus Training and Research Hospital Department of Radiology Ankara Turkey Department of Radiology, Dr. Sami Ulus Training and Research Hospital, Ankara, Turkey; 2 Erzincan Binali Yildirim University Faculty of Medicine Department of Radiology Erzincan Turkey Department of Radiology, Faculty of Medicine, Erzincan Binali Yildirim University, Erzincan, Turkey

**Keywords:** Children, thyroid, nodule, malignancy, location

## Abstract

**Objective:**

As far as we know, in English literature, a limited number of studies has examined the relationship between the location of the nodule and malignancy risk. The studies were performed with adults and their results were mainly inconsistent. We aim to evaluate the potential association between the location of the thyroid nodules and risk for malignancy in the pediatric population.

**Materials and methods:**

Patients younger than 18 years old with a pathological diagnosis were included. Nodules were divided into 5 categories according to the Thyroid Imaging Reporting and Data System (TI-RADS) algorithm. The location of the nodules was recorded: Right lobe, left lobe, isthmus, upper pole, lower pole, and middle. Thyroid glands were divided into 3 equal longitudinal areas to define upper, lower, and middle portions.

**Results:**

Ninety-seven nodules of 103 children were included. The mean age of the population was 14.9 ± 2.51 years (7-18 years). Eighty-one participants were female (83.5%) and 16 male (16.5%). Fifty nodules were benign (51.5%) and 47 nodules were malignant (48.5%). We did not detect a significant correlation between the risk of malignancy and location of the nodule as right or left lobes or isthmus (P = 0.38). Rate of malignant nodules were significantly higher in middle lobe (23%, P = 0.002). Being located at middle part of thyroid gland increases the possibility of malignancy 11.3 times (OR = 11.3, P = 0.006).

**Conclusion:**

Nodule location can be used as a predictor for thyroid malignancy in pediatric patients, similar to adults. Middle lobe location increases the risk of malignancy. Using nodule location along with TI-RADS categorization can increase the efficacy of malignancy prediction.

## INTRODUCTION

Thyroid nodules are common clinical problems that radiologists and clinicians encounter. Thyroid nodules affect approximately 65% of the population and 4-6.5 % of them tend to be malignant. In the pediatric population, the prevalence of thyroid nodules varies between 0.05% and 5.1%, which is less frequent than the prevalence found in adults. However, pediatric thyroid nodules have a greater risk of being malignant; approximately 25% of the nodules can be malignant ( 1 – 3 ).

Ultrasonography is the primary choice for imaging of thyroid nodules. Some ultrasonography characteristics ( *e.g.* , microcalcifications; hypoechogenicity; irregular, microlobulated, or infiltrative margins; and taller-than-wide shape) are widely used for predicting malignancy ( 4 ). Beyond these ultrasonography features, risk stratification systems have also been created. The American College of Radiology developed the Thyroid Imaging Reporting and Data System (TI-RADS) to predict malignancy by using ultrasonography features of the nodules. Studies have found TI-RADS was effective both in adults and children ( 2 , 5 ).

In the literature, examined ultrasonography features generally did not use the location of the nodules as a predictor for malignancy. As far as we know, in the English literature, a limited number of studies has examined the relationship between the location of the nodule and malignancy risk. These studies were performed with adults and their results were mainly inconsistent ( 4 , 6 , 7 ). In the current study, we aim to evaluate the potential association between the locations of the thyroid nodules and risk for malignancy in the pediatric population.

## MATERIALS AND METHODS

This prospective study was approved by the institutional research ethics board, and informed consent was acquired from the patients and their parents.

The study population consisted of 97 nodules of 103 children. Between January 2016 and December 2021, we recorded the sonographic images of the participants. We included patients who were younger than 18 years old and had a pathological diagnosis. The nodules that occupied an entire lobe were excluded (28 patients).

Ultrasonography examinations were performed by using high-frequency linear-array transducers in longitudinal and transverse planes (iU22 by Philips Healthcare and Aplio by Toshiba Medical Systems).

The researchers were not aware of the pathological diagnosis when evaluating ultrasonography features. We primarily reevaluated the ultrasonography images of the patients. Individual evaluations were conducted by 2 radiologists with a combined experience of 8 years and 25 years in pediatric head and neck imaging. If the 2 independent readers disagreed on AE diagnosis, a consensus reading was performed. To minimize recall bias, all cases were evaluated anonymously and randomly in 2 independent sessions 8 weeks apart.

Nodules were divided into 5 categories according to the TI-RADS algorithm (TR1: benign, no need for fine needle aspiration [FNA]; TR2: not suspicious, no need for FNA; TR3: mildly suspicious; TR4: moderately suspicious; and TR5: highly suspicious).

**Figure 1:**
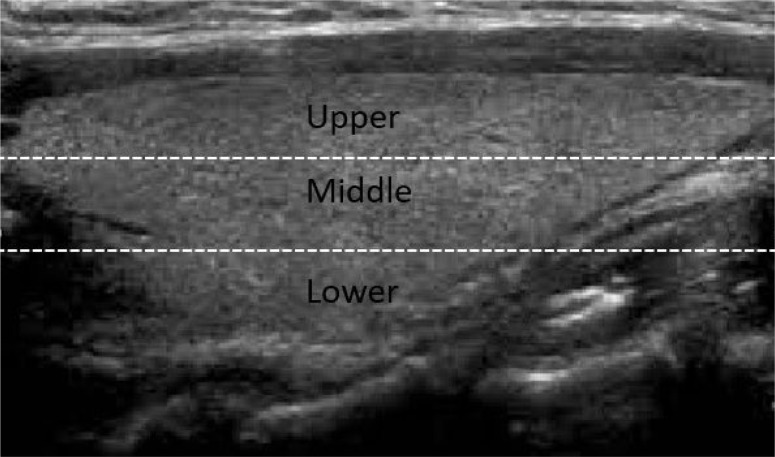
Upper, middle, and lower parts of the thyroid gland.

The location of the nodules was recorded: right lobe, left lobe, isthmus, upper pole, lower pole and middle. Thyroid glands were divided into 3 equal longitudinal areas to define upper, lower, and middle portions ( Figure 1 ). As mentioned, the nodules occupying a whole lobe were excluded. A nodule located in more than 1 longitudinal portion was recorded in the portion where most of the nodule was located.

Nodules were divided into 2 subgroups according to pathology results: benign and malignant. Age and gender of the patients were also recorded.

### Statistical analysis

Data were analyzed using SPSS 20 for Windows (IBM). Normal distribution of the data was evaluated using the Kolmogorov-Smirnov test. Numerical variables with normal distribution were shown as M ± SD. The variables without normal distribution were shown as minimum-maximum and median values. Categorical variables were shown as number and percentage. χ^2^ test was used to compare the percentage of malignancy between different locations. Logistic regression analysis was performed to define the significant risk factors for malignancy. Receiver operating characteristic curve was used to define diagnostic power of TI-RADS categorization. Sensitivity and specificity values were used to compare the diagnostic success of TI-RADS and location data to predict malignancy. Cohen's kappa coefficient was used to determine interrater agreement. Kappa values of agreement were defined as poor between 0.01 and 0.20, fair between 0.21 and 0.40, moderate between 0.41 and 0.60, substantial between 0.61 and 0.80, and nearly perfect between 0.81 and 1.0 ( 8 ).

## RESULTS

Ninety-seven nodules of 103 children were included. Mean age of the population was 14.9 ± 2.51 years old (7-18 years). Eighty-one participants were female (83.5%) and 16 were male (16.5%). Fifty nodules were benign (51.5%) and 47 nodules were malignant (48.5%). There were 13 follicular and 34 papillary carcinoma cases.

The median TI-RADS category of the nodules was TR3. The median TI-RADS category of malignant nodules was significantly higher than the benign ones were (4 *vs.* 2, respectively; *P* = 0.001). The distribution of the nodules according to TI-RADS categories can be seen in Table 1 .

**Table 1:** Number of nodules according to TI-RADS categories

TI-RADS category	Number (percentage)
TR1	15
TR2	17
TR3	44
TR4	13
TR5	8

Forty-three nodules were located at right lobe (44.3%), 43 nodules at left lobe (44.3%), and 11 nodules at isthmus (11.3%). We did not detect a significant correlation between the risk of malignancy and location of the nodule as right or left lobes or isthmus ( *P* = 0.38). Distribution of the benign and malign nodules according to lobes can be seen in Table 2 .

**Table 2:** Benign and malignant nodules according to lobes

Thyroid Lobe	Diagnosis	
	Benign	Malignant
Right	19 (19.6%)	24 (24.7%)
Left	24 (24.7%)	19 (19.6%)
Isthmus	7 (7.2%)	4 (4.1%)

**Figure 2:**
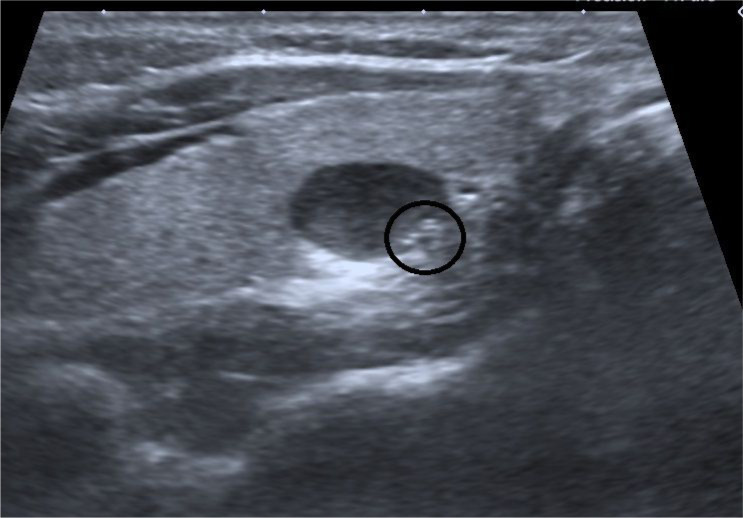
Hypoechoic, solid nodule with eccentric microcalcifications (circle) located at upper pole, right lobe. The pathological diagnosis is papillary cancer.

**Figure 3:**
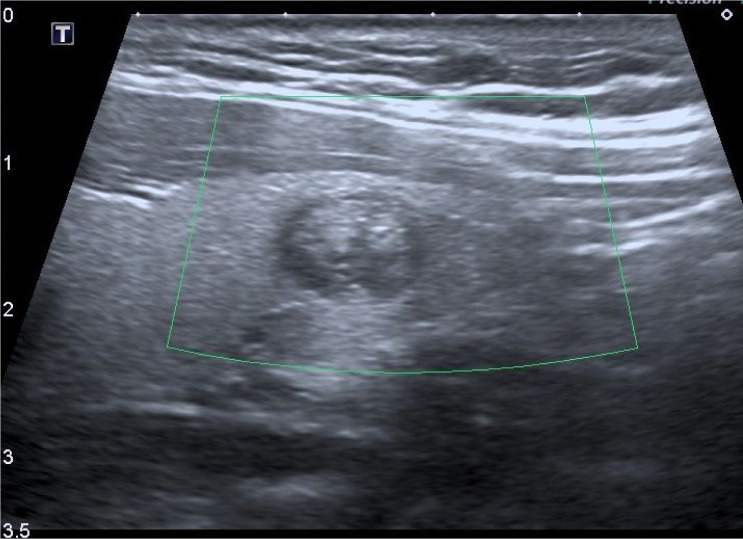
Solid nodule without any vascularization on color Doppler examination, located at the lower pole, right lobe. The pathological diagnosis is a benign colloid nodule.

The majority of the nodules were located at the lower pole (61 nodules, 62.8%). Thirteen nodules were located at the middle (13.4%) and 23 nodules were located at the upper pole (23.7%). The rate of malignant nodules was significantly higher in the middle lobe (23%, *P* = 0.002; Figures 2 and 3 ). Distributions of the benign and malign nodules according to upper, lower, and middle sides of the thyroid can be seen in Table 3 .

**Table 3:** Benign and malignant nodules according to location

Location	Diagnosis	
	Benign	Malignant
Upper pole	20 (86.9%)	3 (13%)
Middle part	10 (76.9%)	3 (23%)
Lower pole	57 (93.4%)	4 (6.5%)

According to logistic regression analysis results, the location of a nodule is a significant, independent risk factor for malignancy. Being located in the middle part of the thyroid gland increases the possibility of malignancy 11.3 times (OR = 11.3, *P* = 0.006). In addition, a 1-point increase in TI-RADS category increases the risk of malignancy 5.78 times (OR = 5.78, *P* = 0.002). The relationship between the middle part location and malignancy remained stable after adjusting for TR3, TR4, and TR5 variables by multivariate regression analysis ( Table 4 ).

**Table 4:** Middle location and the risk of thyroid malignancy adjusted for TI-RADS categories

Variables	Multivariate Regression Results	
	OR	P value
TR3	12.5	0.005
TR4	25.7	0.002
TR5	114.7	0.00

TR: TI-RADS category;

OR: odds ratio.

Having a TI-RADS category of TR3 or higher can predict malignancy with a sensitivity of 78.3% and specificity of 89.8% (area under the curve ± SE = 0.932 ± 0.05). When TI-RADS and location of the nodule are assessed together to predict malignancy, having a nodule located at the middle part of the gland and a category of TR3 or higher can predict malignancy with a sensitivity of 91.7% and specificity of 97.3%.

The kappa value was 0.87 for TI-RADS categorization and 0.92 for nodule localization.

## DISCUSSION

In the English literature, few studies have examined the risk of thyroid malignancy according to nodule location. Studies have also concentrated on adult patients. As far as we know, this is the first study to examine the mentioned relationship in pediatric patients. According to our first and preliminary results, the risk of malignancy is increased for the nodules located in the middle part of the thyroid gland.

A thyroid nodule can be found in approximately 50% of all ultrasonography examinations in the adult population. These nodules are generally asymptomatic and benign. Thyroid cancer affects less than 1% of individuals in their lifetime. Similar to adults, most pediatric thyroid nodules are also benign. However, a thyroid nodule has approximately 3 to 5 times higher risk of malignancy in children. Small tumor size and accurate as well as prompt diagnosis are good prognostic factors for pediatric thyroid cancer ( 9 , 10 ). Therefore, defining suspicious nodules and describing the need for FNA is crucial for fast and accurate diagnosis.

TI-RADS is one of the widely used, ultrasonography-based, risk stratification systems for detecting thyroid malignancy. It was previously validated for adults, and recent studies have shown it can be safely used in pediatric thyroid nodules ( 2 , 11 , 12 ). Our results are consistent with the literature: The median TI-RADS category of malignant nodules was higher than the benign ones were. We also showed that an increase in TI-RADS category significantly increases the risk of malignancy and that the TR3 category can be used as an effective cut-off point to predict malignancy.

We did not detect a significant correlation between malignancy risk and right/left lobe or isthmus locations. Previous results were contradictory: Similar to our results, Ramundo and cols. ( 7 ) stated that malignancy risk did not change according to lobe and isthmus location. However, Jasim and cols. ( 6 ) emphasized that isthmus location increased the risk of malignancy.

In studies, upper lobe nodules were generally found to be more malignant ( 4 , 6 ). Slightly differently, in Ramundo and cols. ( 7 ), middle lobe nodules were defined as having a higher risk of malignancy. No study has defined lower lobe location as a risk factor for malignancy. Our results are in line with the literature. We found that the middle location is an independent risk factor for a thyroid nodule to be malignant. In our study, most of the nodules were located at the lower pole. Results of the previous studies were similar; nodules were generally detected at the lower pole ( 4 , 7 ), except in Jasim and cols. ( 6 ). They stated that most of the included nodules were located at the middle lobe, followed by the lower pole. As stated by previous studies and our results, lower pole nodules are more frequent than the others are, and they generally tend to be benign. Upper pole and middle lobe nodules should be examined more suspiciously for malignancy.

We found that TI-RADS categorization and nodule location had perfect interobserver agreement. This can validate the efficacy of nodule location as a predictor of malignancy.

To define location as a reliable and independent risk factor, we performed multivariate regression analysis with TI-RADS category of the nodules. Our results showed that aside from TI-RADS category, middle lobe location could predict malignancy. Studies conducted in a manner similar to ours have also followed a similar way of analyzing the effectiveness of using location as a predictor. They each used TI-RADS category as an adjustment parameter to test the success of location, and they reached the same conclusion that location can be used as an independent risk factor for malignancy ( 4 , 6 , 7 ). In addition, we found that using TI-RADS categorization and the location of the nodule together increase the sensitivity and specificity of malignancy prediction. Studies have also revealed similar results and have stated that using location along with TI-RADS categorization increases the possibility of defining a malignant nodule ( 6 , 7 ).

This study has limitations to be mentioned. The retrospective nature of the study creates a selection bias. The number of the nodules can be increased for results that are more reliable. Large nodules, occupying a whole lobe, were excluded; therefore, we cannot offer any information about these kinds of nodules. We lacked an anatomic landmark to describe the upper, middle, and lower portions of the thyroid gland, but in our view, a perfect interobserver agreement value largely overcomes this limitation.

In conclusion, nodule location can be used as a predictor for thyroid malignancy in pediatric patients, similar to adults. Middle lobe location increases the risk of malignancy. Using nodule location along with TI-RADS categorization can increase the efficacy of malignancy prediction.
